# The complete mitochondrial genome of *Lophosquillia costata* (Malacostraca: Stomatopoda) from China and phylogeny of stomatopods

**DOI:** 10.1080/23802359.2020.1780170

**Published:** 2020-06-19

**Authors:** Yazhou Zhang, Yuanxin Bi, Meiping Feng

**Affiliations:** a Marine Fisheries Research Institute of Zhejiang Province, Zhoushan, China; b Scientific Observing and Experimental Station of Fishery Resources for Key Fishing Grounds, Zhoushan, China; c Key Laboratory of Sustainable Utilization of Technology Research for Fishery Resources of Zhejiang Province, Zhoushan, China; d Engineering Technology Research Center of Marine Ranching, College of Marine Ecology and Environment, Shanghai Ocean University, Shanghai, China

**Keywords:** *Lophosquillia costata*, mitochondrial genome, Stomatopoda, phylogeny

## Abstract

Here, we present the complete mitochondrial genome of *Lophosquillia costata*. The genome is 15,771 bp in length with a 68.07% AT content. It contains 13 protein-coding genes, two rRNAs genes, and 22 tRNAs. Both rRNAs are encoded on the light strand. Besides seven tRNAs are encoded on the light strand (trnY, trnQ, trnV, trnL1, trnP, trnH, and trnF), and four PCG (nad1, nad4l, nad4, and nad5) are encoded on the light strand, whereas the other nine PCGs are located on the heavy strand. Phylogenetic analysis based on mitochondrial PCGs shows two distinct groups for Stomatopoda and Decapoda. *Lophosquillia costata* is found clustered with *Oratosquilla oratoria* into a branch (BP = 100), and they grouped with other species with high support (BP = 99) in the family Squillidae. Our results shall provide a better understanding in the evolutionary histories of the stomatopods.

Stomatopods form an important component of marine ecosystems and are economically significant (Van Der Wal et al. 2019). The largest superfamily the order of Stomatopoda is Squilloidea, which is morphologically diverse and includes over 185 species in 49 genera (Ahyong 2001, 2005; Van Der Wal and Ahyong 2017). As active predators in muddy and sandy substrates on coastal and continental shelf habitats (Abelló and Martin 1993; Ahyong 2005), some species of Squilloidea are also major fisheries targets, including *Squilla mantis* (Abelló and Martin 1993; Maynou et al. 2004), *Oratosquilla oratoria* (Zhang et al. 2012) and *Harpiosquilla raphidea* (Wardiatno and Mashar 2010). Studies on Squilloidea have been focused on *Oratosquilla oratoria* in China (Zhang et al. 2012, 2016; Yang and Li 2018), and here we report on another species, *Lophosquilla costata* (de Haan, 1844) (Crustacea: Malacostraca: Stomatopoda: Squillidae), one of the dominant species of mantis shrimps in the Zhoushan Fishing Ground and its adjacent waters (Yu et al. 2011).

As of 15 March 2020, GenBank contained seven complete mitochondrial genomes of Stomatopoda, including three families. There is one partial mitochondrial sequence (GenBank: MH168237.1) of *Lophosquilla costata* in Genbank, however, the whole mitochondrial genome is still in lack. Here, we present the first complete mitochondrial genome of the *L*. *costata*.

Specimens of *L*. *costata* were collected from Dongfushan Island (30.13°N, 122.77°E), Zhoushan archipelago, in the East China Sea. The muscle tissue isolated from the fresh specimen was immediately preserved in 95% ethanol and kept in −80 °C. DNA was extracted with E.Z.N.A^®^DNA kit (OMEGA, USA), and mitochondrial DNA was amplified with a DNA REPLI-g Mitochondrial DNA Kit (QIAGEN, Hilden, Germany) as directed by the manufacturer. Library construction and sequencing were performed by Biozeron (Biozeron, Shanghai, China) using the Illumina HiSeq 4000 sequencing platform (Illumina, San Diego, CA). The specimen is stored in −80 °C in Fishery resources lab in Marine and Fisheries Research Institute of Zhejiang Province (Mshrimp MT-1).

The mitochondrial genome of *L*. *costata* is a circular molecule which is 15,771 bp (GenBank accession number: MT276143) in length. It contains 13 protein-coding genes (PCGs), two rRNAs genes, and 22 tRNAs. Total AT content of *L*. *costata* is 68.07%. Both rRNAs are encoded on the light strand. Besides seven tRNAs are encoded on the light strand (trnY, trnQ, trnV, trnL1, trnP, trnH, and trnF), and four PCG (nad1, nad4l, nad4, and nad5) are encoded on the light strand, whereas the other nine PCGs are located on the heavy strand.

To elucidate phylogenetic relationships of *L*. *costata* with the other stomatopods, phylogenetic tree (Figure 1) is constructed based on the PCGs with Maximum-Likelihood using phyML ver 3.0 (http://www.atgc-montpellier.fr/phyml/). The genetic information from Order Stomatopoda has only been reported several times (e.g. Kundu et al. 2018), those from Order Decapoda are also considered (e.g. Sung et al. 2018). A total of six mitochondrial genomes from Stomatopoda and 12 mitochondrial genomes from Decapoda have been used in the phylogenetic tree. All the six species from Order Stomatopoda were belonged to the same family except *Lysiosquillina maculata* (Lysiosquillidae) and *Gonodactylus chiragra* (Gonodactylidae). Results show two distinct groups for Stomatopoda and Decapoda. *Lophosquillia costata* is found clustered with *Oratosquilla oratoria* into a branch (BP = 100), and they grouped with other species with high support (BP = 99) in the family Squillidae. Two species from other families, *Lysiosquillina maculata* and *Gonodactylus chiragra*, are the most distantly related species within Stomatopoda, also with high support (BP = 99).

**Figure 1.**
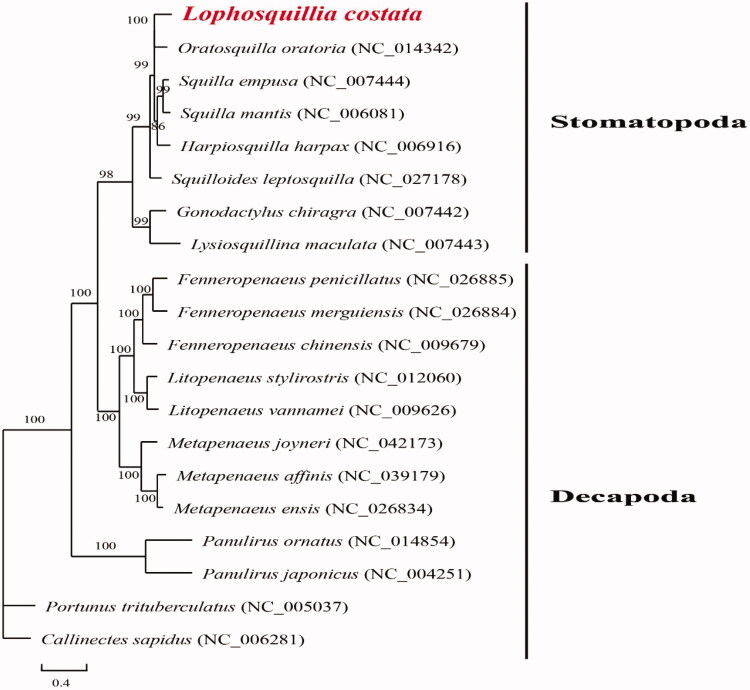
Phylogenetic tree of *Lophosquillia costata* and other genomes from Order Stomatopoda and Decapoda based on mitochondrial PCGs.

In this study, we present the complete mitochondrial genome sequence of *L*. *costata*, which would contribute to further phylogenetic analysis of this species. Furthermore, more mitochondrial genomic data of undetermined taxa and further analysis are required to reveal phylogeny and evolution of stomatopods.

## Data Availability

The data that support the findings of this study are openly available in the Genbank database at https://www.ncbi.nlm.nih.gov/ (Accession number: MT276143) after 31 May 2021, and also available from the corresponding author [YX. Bi], upon reasonable request.
